# A novel hypoxia- and lactate metabolism-related signature to predict prognosis and immunotherapy responses for breast cancer by integrating machine learning and bioinformatic analyses

**DOI:** 10.3389/fimmu.2022.998140

**Published:** 2022-10-07

**Authors:** Jia Li, Hao Qiao, Fei Wu, Shiyu Sun, Cong Feng, Chaofan Li, Wanjun Yan, Wei Lv, Huizi Wu, Mengjie Liu, Xi Chen, Xuan Liu, Weiwei Wang, Yifan Cai, Yu Zhang, Zhangjian Zhou, Yinbin Zhang, Shuqun Zhang

**Affiliations:** ^1^ Department of Oncology, The Second Affiliated Hospital of Xi’an Jiaotong University, Xi’an, China; ^2^ Department of Orthopedics, The Second Affiliated Hospital of Xi’an Jiaotong University, Xi’an, China

**Keywords:** breast cancer, hypoxia, lactate metabolism, immunotherapy, immune microenvironment (IME), machine learning, bioinformatics

## Abstract

**Background:**

Breast cancer is the most common cancer worldwide. Hypoxia and lactate metabolism are hallmarks of cancer. This study aimed to construct a novel hypoxia- and lactate metabolism-related gene signature to predict the survival, immune microenvironment, and treatment response of breast cancer patients.

**Methods:**

RNA-seq and clinical data of breast cancer from The Cancer Genome Atlas database and Gene Expression Omnibus were downloaded. Hypoxia- and lactate metabolism-related genes were collected from publicly available data sources. The differentially expressed genes were identified using the “edgeR” R package. Univariate Cox regression, random survival forest (RSF), and stepwise multivariate Cox regression analyses were performed to construct the hypoxia-lactate metabolism-related prognostic model (HLMRPM). Further analyses, including functional enrichment, ESTIMATE, CIBERSORTx, Immune Cell Abundance Identifier (ImmuCellAI), TIDE, immunophenoscore (IPS), pRRophetic, and CellMiner, were performed to analyze immune status and treatment responses.

**Results:**

We identified 181 differentially expressed hypoxia-lactate metabolism-related genes (HLMRGs), 24 of which were valuable prognostic genes. Using RSF and stepwise multivariate Cox regression analysis, five HLMRGs were included to establish the HLMRPM. According to the medium-risk score, patients were divided into high- and low-risk groups. Patients in the high-risk group had a worse prognosis than those in the low-risk group (*P* < 0.05). A nomogram was further built to predict overall survival (OS). Functional enrichment analyses showed that the low-risk group was enriched with immune-related pathways, such as antigen processing and presentation and cytokine-cytokine receptor interaction, whereas the high-risk group was enriched in mTOR and Wnt signaling pathways. CIBERSORTx and ImmuCellAI showed that the low-risk group had abundant anti-tumor immune cells, whereas in the high-risk group, immunosuppressive cells were dominant. Independent immunotherapy datasets (IMvigor210 and GSE78220), TIDE, IPS and pRRophetic analyses revealed that the low-risk group responded better to common immunotherapy and chemotherapy drugs.

**Conclusions:**

We constructed a novel prognostic signature combining lactate metabolism and hypoxia to predict OS, immune status, and treatment response of patients with breast cancer, providing a viewpoint for individualized treatment.

## Introduction

Breast cancer (BC) is the most common cancer among women, with an annual incidence rate of 0.5% (1). Developing comprehensive treatment strategies has significantly improved outcomes for patients with BC. Immune checkpoint inhibitors (ICI) are revolutionizing cancer treatment but are relatively restricted to the triple-negative histological subtype (2). However, many BC patients have poor outcomes owing to recurrence, metastasis, and chemotherapy resistance (3). It is still challenging to develop effective biomarkers to identify patients with BC and poor prognoses while guiding treatment.

Hypoxia is a typical tumor microenvironment (TME) feature in nearly all solid tumors. Many features of cancer can cause hypoxia, including uncontrolled tumor proliferation, tumor micro-vessel abnormalities, diffusion geometry deterioration, and tumor-associated anemia (4, 5). Extensive reviews have shown that hypoxia can regulate tumor proliferation, angiogenesis, aggressiveness, metastasis, and radiotherapy resistance (6–8). Hypoxia can also influence genetic instability, proteomic changes, genetic hypoxia-resistance, and stem cell phenotype maintenance (9–13). Moreover, hypoxia can profoundly impact large-scale proteomic changes *via* several transcription factors, especially hypoxia-inducible factor 1 [HIF-1] (14). A significant portion of intratumoral lactate accumulation can also be induced by HIF-1-mediated metabolic reprogramming (15).

Lactate is the product of anaerobic glycolysis. It is closely related to the development, maintenance, progression, TME, metastasis, and treatment resistance of cancers (16–18). Lactate accumulation in intratumoral tissue is primarily a consequence of HIF-1-mediated metabolic reprogramming; however, several HIF-1-independent mechanisms also produce lactate, such as MYC activation (19). Alterations in lactate metabolism are associated with cell invasion, migration, angiogenesis, drug resistance, and immune escape. In gastric cancer, abnormal lactate metabolism can lead to acquired resistance *via* the NF-κB pathway (20). In BC, lactate generated by tumor cells induces programmed death-ligand 1 (PD-L1) in tumor cells, causing tumor-specific antigens to evade immune cells, thereby promoting growth (21). Enhanced lactate exposure can affect the phenotype of MCF7 cells and promote tamoxifen resistance (22). Furthermore, high lactate levels could increase cancer stemness and lead to worse clinical outcomes in patients with BC (23).

As the main features of TME, both hypoxia and lactate can regulate the anti-tumor immune response. Hypoxia attenuates anti-tumor immunity by increasing the pro-tumorigenic M2 phenotype, intratumoral accumulation of immunosuppressive regulatory T cells, and stimulation of adenosine receptors (12, 24, 25). Lactate inhibits CD8+ and CD4+ effector T cell function but increases T helper 1 cell differentiation and interferon-γ (IFNγ) production (26–30). Hypoxia can also upregulate PD-L1 by binding HIF-1 to the hypoxia response element in the PD-L1 proximal promoter (31, 32). Recent research reported that lactate could regulate programmed cell death protein 1 (PD-1) specifically in effector regulatory T (eTreg) cells, thereby leading to treatment failure of ICIs; suppression of lactate metabolism in Treg cells enhances sensitivity to ICIs in resistant tumors (33).

The role of hypoxia and lactate metabolism in diverse cancers has been further demonstrated by genomic studies in recent years. The effect of hypoxia on prognosis, treatment guidance, and immune infiltration assessment has been reported in many tumors, such as cervical cancer, head and neck squamous cell carcinoma, and BC (34–36). Simultaneously, lactate metabolism was related to the outcomes and immune microenvironment in skin cutaneous melanoma, kidney renal clear cell carcinoma, and BC (37–39). In BC, prognostic models have been developed solely using lactate metabolism-related genes (LMRGs) or hypoxia-related genes [HRGs] (39–41). However, considering the heterogeneity of BC and the complex interactions between hypoxia and lactate metabolism, these alone cannot fully identify the relevant characteristics of BC. Hence, landscape assessment of the fundamental combination of hypoxia and lactate metabolism on BC prognosis, TME, and ICIs therapy remains necessary.

In this study, we simultaneously considered the impact of lactate metabolism and hypoxia on BC by applying both LMRGs and HRGs in constructing a hypoxia-lactate metabolism-related prognostic model (HLMRPM) that could accurately predict BC prognoses, immune status, and therapy response.

## Methods

### Data collection

RNA sequencing and clinical data of 1113 BC cases were obtained from The Cancer Genome Atlas (TCGA) data portal, along with 113 normal tissue samples (https://portal.gdc.cancer.gov/). The University of California Santa Cruz (UCSC) Xena provided data on genotype-tissue expression [GTEx] (https://xenabrowser.net/datapages/), a comprehensive public resource for studying normal tissue-specific gene expression and regulation. We retained 1033 patients with overall survival (OS) time longer than 30 days. The microarray dataset GSE20685 (N = 327) was downloaded from the Gene Expression Omnibus database (GEO, http://www.ncbi.nlm.nih.gov/geo/). For immunotherapy response predictions, two immunotherapeutic cohorts were included in our study: The IMvigor210 cohort (advanced urothelial cancer with atezolizumab intervention) was downloaded from the website based on the Creative Commons 3.0 license (http://research-pub.Gene.com/imvigor210corebiologies) (42); the GSE78220 (metastatic melanoma with pembrolizumab treatment) was downloaded from the GEO (43). The main abbreviations were list in **Table S1**.

The predefined gene sets included in our research were acquired from the Molecular Signatures Database (MSigDB; https://www.gsea-msigdb.org/gsea/msigdb/index.jsp) (44). We used the terms “lactic” and “hypoxia” as the search keywords in the MSigDB database. Five priority LMRG sets were eventually determined: GOBP lactate metabolic process, HP increased serum lactate, HP lactic acidosis, HP lactic aciduria, and HP severe lactic acidosis. Seven priority HRG sets were eventually determined: hallmark hypoxia, winter hypoxia metagene, harris hypoxia, Buffa hypoxia, Mizukami hypoxia down, Mizukami hypoxia up, and reactome cellular response to hypoxia. After deleting duplicates, 284 LMRGs and 493 HRGs were identified for subsequent analysis [**Table S2**].

### Identification of differentially expressed genes, differentially expressed LMRGs and differentially expressed HRGs

We analyzed differentially expressed genes (DEGs) using the R package “edgeR” in R (| log_2_ fold change [FC]|>1 and *P* < 0.05). We then identified differentially expressed LMRGs (DELMRGs) and HRGs (DEHRGs) by intersecting DEGs with LMRGs and HRGs, and visualizing them using the Venn diagram with the R package “VennDiagram”. The relationship between DELMRGs and DEHRGs in BC was assessed using the R package “corrr”. The protein-protein interaction (PPI) network was constructed using the STRING database (https://string-db.org/) and Cytoscape (v3.9.0). The web-based tool Metascape (http://metascape.org/) could perform gene annotation and functional enrichment analysis and be used for annotating DELMRGs and DEHRGs.

### Construction of hypoxia-lactate metabolism-related prognostic model

Using Cox regression analysis, prognostic DEHRGs and DELMRGs were identified (*P* < 0.05). We further divided the prognostic genes into favorable genes, where high RNA expression correlates with longer survival time, and unfavorable genes, where high RNA expression correlates with shorter survival times. To construct a robust HLMRPM, we used the random survival forest (RSF) algorithm to reduce the dimensions of genes using the R packages “randomForestSRC” and “randomSurvivalForest” (45). RSF is a non-parametric tree-based ensemble learning method that can automatically select and rank variables (46, 47). Genes ranked in the top 15 lists of variable importance (VIMP) and minimal depth were reserved as the most important prognostic hypoxia-lactate metabolism-related genes (HLMRGs). The stepwise multivariate Cox regression analysis constructed the HLMRPM [risk score = (0.6139585 ×ESRP1) + (-0.3698120 ×MAFF) + (0.1682696×SLC2A1) + (-0.2963183 × DARS2) + (0.2690044 ×TH)]. Each patient had a risk score and was grouped into a high- or low-risk group based on the medium-risk score.

Furthermore, the prognostic prediction value of the model was investigated in the TCGA and GSE20685 cohorts. Kaplan–Meier survival analysis was performed using the R package “survminer” to compare OS between the two risk groups. Receiver operating characteristic (ROC) curves were constructed using the R package “timeROC” to assess prediction efficiency. Finally, univariate and multivariate Cox regression analyses were done to determine the independent prognostic value of the HLMRPM.

### Stratified analysis and independent prognostic analysis

Stratified analysis was used to assess the prognostic value of HLMRPM in different subgroups stratified by clinical features. We assessed HLMRPM accuracy using ROC curves. We performed univariate and multivariate Cox regression analyses with the risk score, age, stage, and T, N, and M stages to evaluate the independent prognostic factors for BC.

### Construction of the prognostic nomogram

To calculate the 1-, 3-, and 5-year OS probabilities, a nomogram was constructed using independent prognostic factors. ROC curves, C-index, and calibration curves were used to evaluate the performance of the nomogram. We further measured the net benefit of the nomogram and clinical features alone with decision curve analysis (DCA).

### Functional enrichment analysis

To analyze the typical functional features of the two risk groups, we analyzed the DEGs between the two groups and then annotated them with Gene Ontology (GO) and Kyoto Encyclopedia of Genes and Genomes (KEGG) using the R package “ClusterProfiler” (48). Gene set enrichment analysis (GSEA) was also used to explore variations in pathway activities between the two risk groups (*P* < 0.05 and false discovery rate (FDR) < 0.25) (49). Annotated gene sets “c2.cp.kegg.v7.5.1. symbols.gmt” were downloaded from MSigDB. We further used the R package “ClusterProfiler” to visualize the results.

### The correlations between the risk score and stem cell-like features

Tumour stemness can be measured with RNA stemness score based on mRNA expression (RNAss) and DNA stemness score based on DNA methylation pattern (DNAss) (50). Spearman correlation test was used to examine the association between the risk score and RNAss.

### Assessment of the TME, immune cell infiltration, and immunotherapy response

The immune cell abundance identifier (ImmuCellAI) can predict the response to ICIs by assessing the abundance of immune cells, especially different T cell subsets (51). ImmuCellAI was used to assess the abundance of infiltrating immune cells according to the “ssGSEA” algorithm. Furthermore, we used the ESTIMATE algorithm with the R package “estimate” to assess the proportions of TME components (52), resulting in four indices: tumor purity, immune, stromal, and ESTIMATE scores. A higher score indicates a larger proportion of components in the TME. We further performed the “Cibersort” algorithm to analyze the infiltration levels of immune cell types (53).

We compared the expression of well-known immune checkpoint genes (ICGs) between the two risk groups. Immunophenoscore (IPS) is a machine-learning-based system that calculates z-scores based on four immunogenicity-related cell types (54). IPS and an online tool called TIDE were used to predict patient response to ICIs (http://tide.dfci.harvard.edu/) (55, 56).

The IMvigor210 and GSE78220 cohorts were further used to validate the predictive power of the HLMRPM for ICIs response. Patients who achieved complete remission (CR) or partial response (PR) or stale disease (SD) were classified as responders and compared with non-responders who showed signs of progressive disease (PD).

### Assessment of the sensitivity to chemotherapy drugs in two risk groups

To assess the association between the risk score and drug sensitivity, we used the R package “pRRophetic” and the CellMiner database. The R package “pRRophetic” was used to calculate the half-maximal inhibitory concentrations (IC50) of common chemotherapy drugs (57, 58). Wilcoxon signed-rank tests were used to compare IC50 values between the two risk groups. We further predicted the potential target drugs (approved by the FDA and those in clinical tests) that could target the five HLMRGs in the HLMRPM using the CellMiner database (https://discover.nci.nih.gov/cellminer) (59, 60).

### Verification of five HLMRGs in databases

We verified HLMRG expression using other online public databases. We analyzed HLMRG expression in BC tissues and normal tissues from TCGA and GTEx. In addition, we performed a survival analysis of five HLMRGs in the TCGA cohort. We further assessed immunohistochemical images and staining intensity of HLMRGs in BC and normal tissues from the Human Protein Atlas (HPA) database (https://www.proteinatlas.org/). In the HPA database, four categories of high, medium, low, and not detected were used to evaluate expression levels. These categories included a scoring system based on the proportion of positive-stained cells (>75, 25–75, or <25%) and staining intensity (strong, moderate, weak, or negative). We further aggregated the staining intensities of the five HLMRGs in breast cancer and normal tissues from the HPA database. The biological functions of the five HLMRGs were assessed using the Gene Set Cancer Analysis (GSCA) database (http://bioinfo.life.hust.edu.cn/GSCA/#/). We also evaluated the association of these five genes with immune cell infiltration.

### Statistical analysis

Statistical analyses were performed using the R software (version 4.0.5). The Wilcoxon signed-rank test was used to compare the differences between the two groups. All tests were two-sided; a p-value of less than 0.05 was considered statistically significant; and the significance levels were set at * *P* ≤ 0.05, ** *P* ≤ 0.01, and *** *P*≤ 0.001.

## Results

### Identification of DEGs, DELMRGs, and DEHRGs in breast cancer

**Figure 1:**
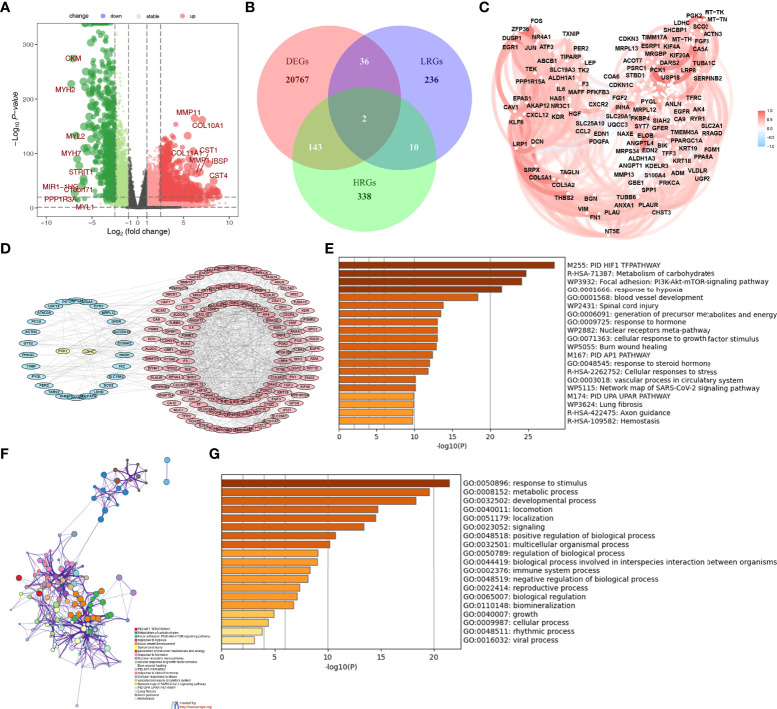
Identification of the differential expressed HRGs and LMRGs. **(A)** The volcano plot of the DEGs. **(B)** The Veen diagram of the DEGs, HRGs, and LMRGs. **(C)** The relationship of the DEHRGs and DELMRGs in breast cancer. **(D)** The PPI network of the DEHRGs and DELMRGs in the string database. **(E–G)** The function of the DEHRGs and DELMRGs in the Metascape database.

**Figure 2:**
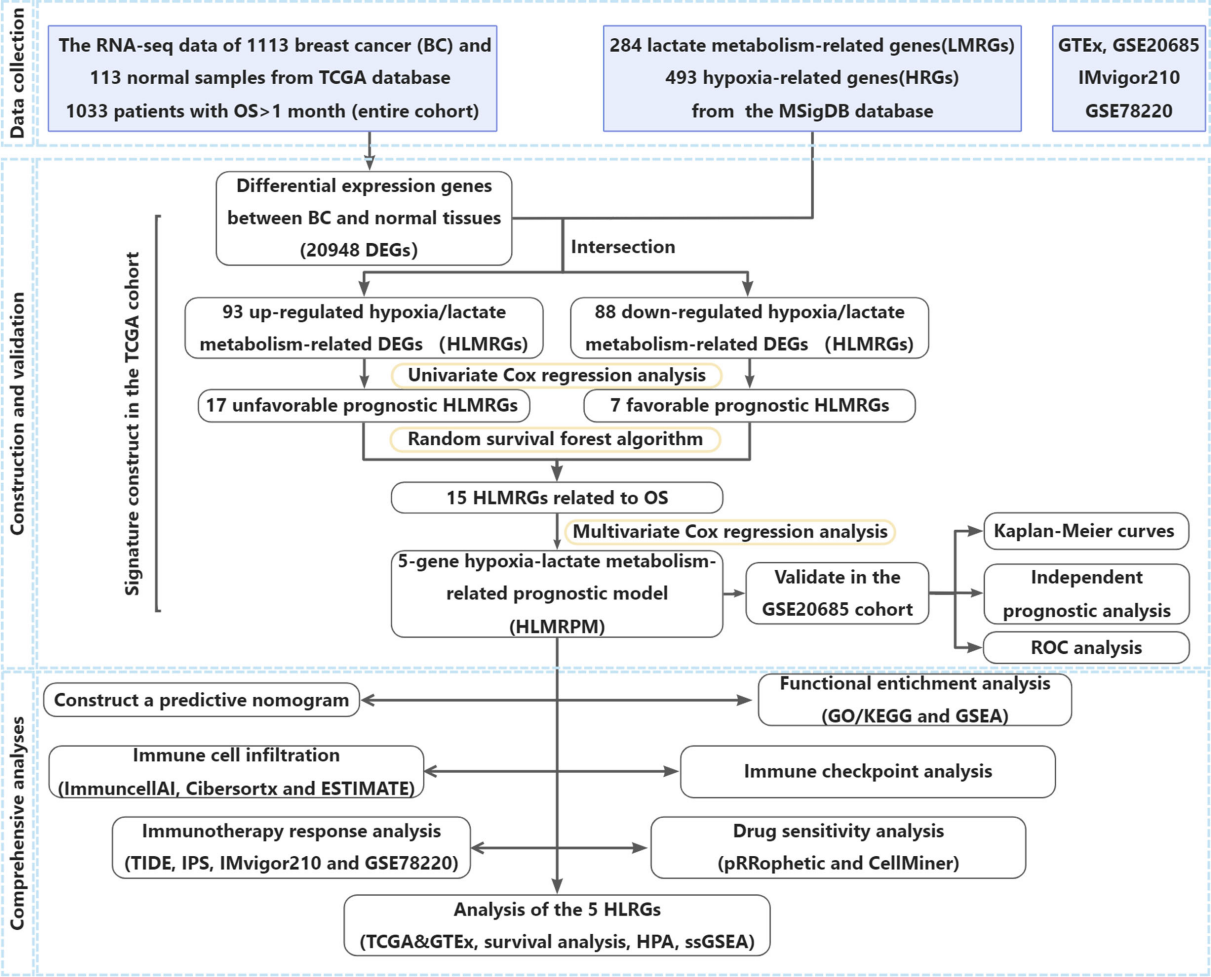
The flow of the study.

There were 20948 DEGs in BC compared with normal tissues, including 17466 upregulated and 3482 downregulated genes. The volcano plot showed 20948 DEGs (**Figure 1A**). The Venn diagram showed that there were 145 DEHRGs and 38 DELMRGs, two of the genes were shared (**Figure 1B**). **Figure 1C** showed a strong positive correlation between DEHRGs and DELMRGs in BC. The PPI network revealed intrinsic correlations between the DEHRGs and DELMRGs (**Figure 1D**). Functional enrichment analyses in the Metascape database revealed that DEHRGs and DELMRGs were closely associated with hypoxia and metabolic processes (**Figures 1E–G**). The workflow of this study is illustrated in **Figure 2**.

### Construction of the HLMRPM and assessment of its predictive ability

**Figure 3:**
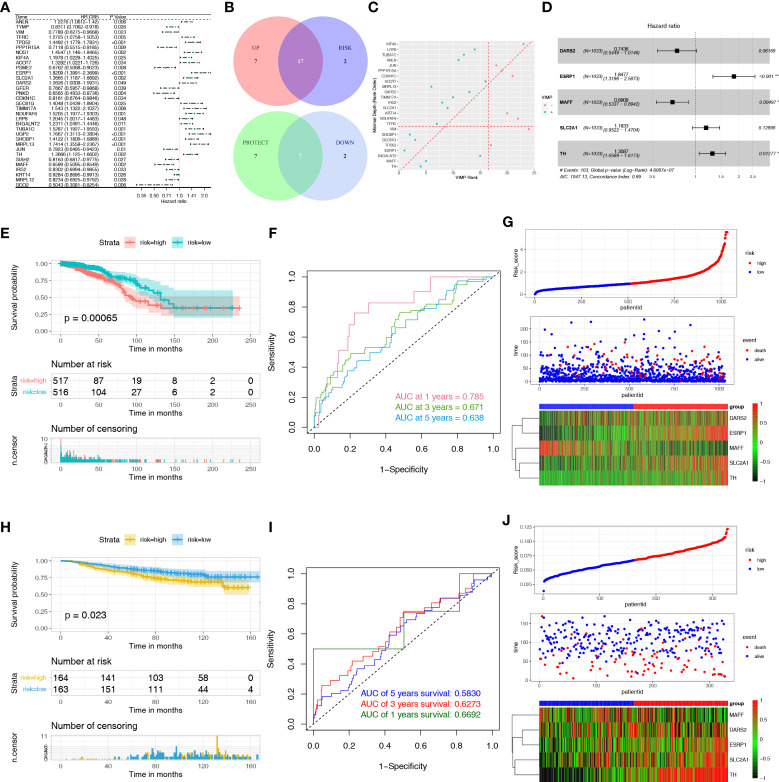
Construction and evaluation of the HLMRPM. **(A)** Univariate Cox regression analysis of the DEHRGs and DELMRGs in the training cohort. **(B)** The veen diagram indicated the favorable and unfavorable genes. **(C)** The genes were ranked by the minimal depth and VIMP. **(D)** The forest graph showed the results of stepwise multivariable cox proportional hazards regression analysis. **(E)** The OS curve of the two risk groups in TCGA cohort. **(F)** The time-dependent ROC curves of the HLMRPM in TCGA cohort. **(G)** The risk score, clinical event, and the model genes between the two risk groups in TCGA cohort. **(H)** The OS curve of the two risk groups in GSE20685 cohort. **(I)** The time-dependent ROC curves of the HLMRPM in GSE20685 cohort. **(J)** The risk score, clinical event, and the model genes between the two risk groups in GSE20685 cohort.

To identify DEHRGs and DELMRGs with prognostic value, we performed a univariate Cox regression analysis and obtained 33 DEGs with significant effects on patient prognosis, including 7 DELMRGs and 26 DEHRGs (**Figure 3A**). Seventeen unfavorable DEGs with HR > 1 in breast cancer and seven favorable DEGs with HR < 1 in breast cancer were used for further analysis (**Figure 3B**). Finally, 15 genes (ACOT7, B4GALNT2, CDKN1C, DARS2, ESRP1, IRS2, MAFF, MRPL13, SEC61G, SHCBP1, SLC2A1, TFRC, TH, TIMM17A, and VIM) were retained and ranked in the top 15 by the minimal depth and VIMP (**Figure 3C**). Using multivariate Cox regression, the five genes comprise the HLMRPM: risk score = (0.6139585 × ESRP1) + (-0.3698120 × MAFF) + (0.1682696 × SLC2A1) + (-0.2963183 × DARS2) + (0.2690044 ×TH) (**Figure 3D** and **Table S3**). Patients were then categorized into high- and low-risk groups based on their median risk scores. The high-risk patients suffered from poor outcomes (**Figure 3E**). The area under curves (AUCs) of HLMRPM in predicting the 1-, 3-, and 5-year OS were 0.785, 0.671, and 0.638 in the TCGA cohort (**Figure 3F**). The risk score, clinical events, and five HLMRG expressions between the two risk groups were illustrated in **Figure 3G**.

In the GSE20685 cohort, the high-risk patients suffered poor outcomes (**Figure 3H**). The 1-, 3-, and 5-year AUCs were 0.583, 0.627, and 0.669, respectively (**Figure 3I**). The risk score, clinical events, and five HLMRG expressions between the two risk groups were similar to the TCGA cohort (**Figure 3J**).

### Stratified analysis and independent prognostic analysis

**Figure 4:**
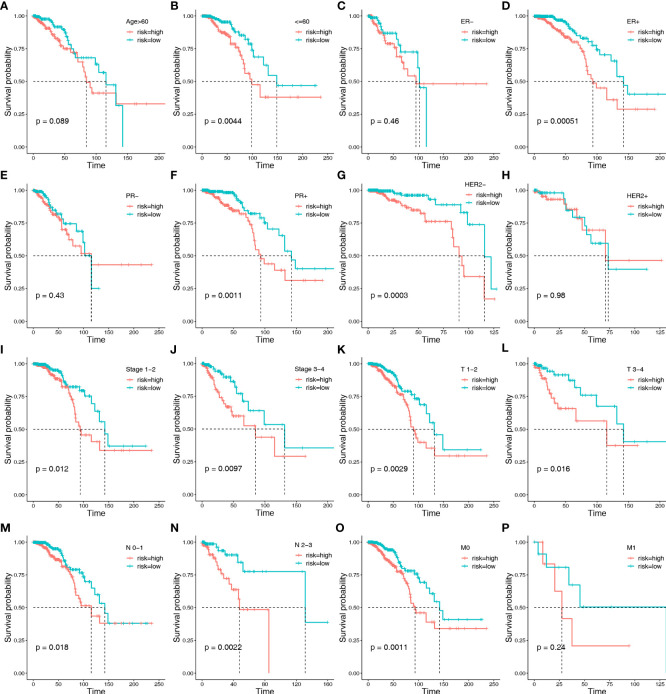
Kaplan–Meier survival analysis between the two risk groups in subgroups stratified by clinical characteristics: including age [>60 years vs. ≤60 years] **(A, B)**, ER stage [negative vs. positive] **(C, D)**, HER2 stage [negative vs. positive] **(E, F)**, PR stage [negative vs. positive] **(G, H)**, stages [stage1-2 vs. stage3-4] **(I, J)**, AJCC T stage [T1-2 vs. T3-4] **(K, L)**, AJCC N stage [N0-1 vs. T2-3] **(M, N)**, and AJCC M stage [M0 vs. M1] **(O, P)**, respectively.

To further verify the ability of HLMRPM to accurately and independently predict the outcome of patients with BC, we performed stratification analysis, Cox regression analysis, and ROC curves. We assigned patients to different subgroups according to age (>60 vs. ≤60 years), ER stage (negative vs. positive), HER2 stage (negative vs. positive), PR stage (negative vs. positive), stages (stage1-2vs. stage3-4), American Joint Committee on Cancer (AJCC) T stage (T1-2 vs. T3-4), AJCC N stage (N0-1 vs. T2-3), and AJCC M stage (M0 vs. M1). We then conducted Kaplan–Meier survival analysis. We found that high-risk patients consistently showed significantly worse outcomes in many subgroups, including age ≤60 years, ER-positive, PR-positive, HER2-negative, stage1-2, stage3-4, T1-2, T3-4, N0-1, N2-3, and M0 stages (**Figures 4A–P**). These results demonstrated the universal applicability of the HLMRPM.

**Figure 5:**
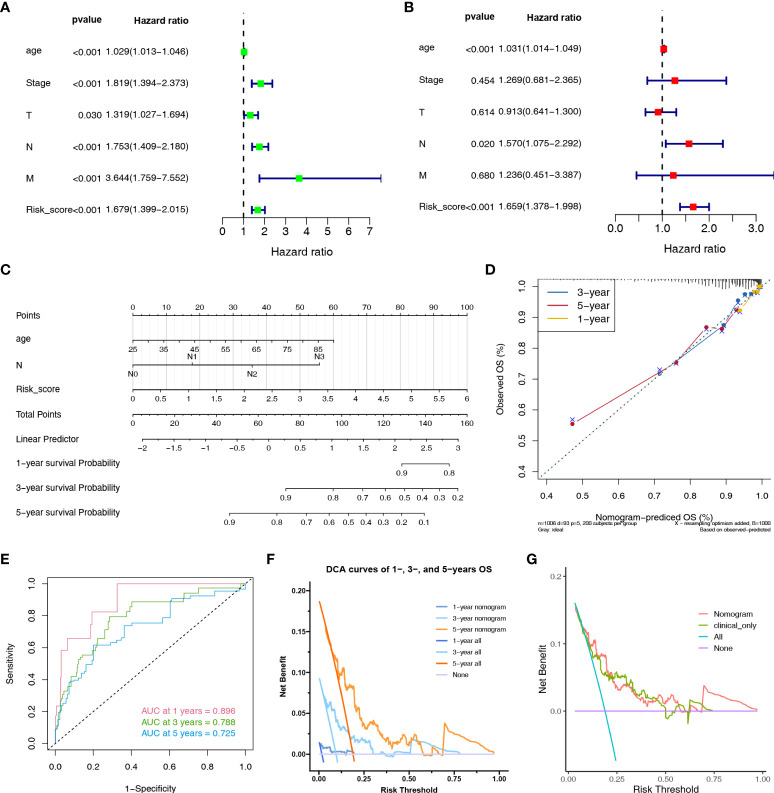
Nomogram to evaluate the OS probability of BC patients. The univariate **(A)** and multivariate **(B)** Cox regression analyses in TCGA cohort. **(C)** The nomogram for predicting the 1-, 3- and 5-year OS probabilities. **(D)** Calibration curves of the nomogram to predict 1-, 3- and 5-year OS probabilities. **(E)** ROC curves of the nomogram. **(F)** DCA curves of the nomogram at 1-, 3- and 5-year indicated its net clinical benefits. **(G)** DCA curves of the nomogram and only clinical characteristics (combination of age and N stage).

Furthermore, we performed univariate and multivariate Cox regression analyses on risk score, age, stage, T, N, and M stage in the TCGA cohort. Based on univariate Cox regression analysis, the hazard ratio (HR) and 95% confidence interval (CI) of the risk score, age, stage, T, N, and M stage were 1.679 (1.399–2.015), 1.029 (1.013–1.046), 1.029 (1.013–1.046), 1.819 (1.394-2.373), 1.753 (1.409–2.180), and 3.644 (1.759–7.552), respectively (*P* < 0.05) (**Figure 5A**). After multivariate Cox regression analysis, the HR and 95% CI of the risk score, age and N stage were 1.659 (1.378–1.998), 1.031 (1.014–1.049), and 1.570 (1.075–2.292) (*P* < 0.05), respectively (**Figure 5B**).

### Establishment of a predictive nomogram

Clinical nomograms are widely used in predicting patient survival by computing set points based on nomogram scores. A nomogram was constructed based on independent prognostic markers (risk score, age, and N stage) to quantitatively predict the 1-, 3-, and 5-year OS rates in patients with BC (**Figure 5C**). The C-index of the nomogram is 0.794. The calibration curves for the nomogram showed an ideal prediction accuracy (**Figure 5D**). The AUC values of the nomogram were 0.896, 0.788, and 0.725 at 1, 3, and 5-year, respectively (**Figure 5E**). The DCA curve was used to render clinical validity to the nomograms (61). **Figure 5F** demonstrated that the nomogram could provide many short- and long-term net clinical benefits. **Figure 5G** demonstrated that the nomogram could bring more net clinical benefits than clinical characteristics alone. Taken together, the nomogram based on HLMRPM could predict both short- and long-term OS in patients with BC, which could assist in clinical management.

### Functional enrichment analyses of the two risk groups

**Figure 6:**
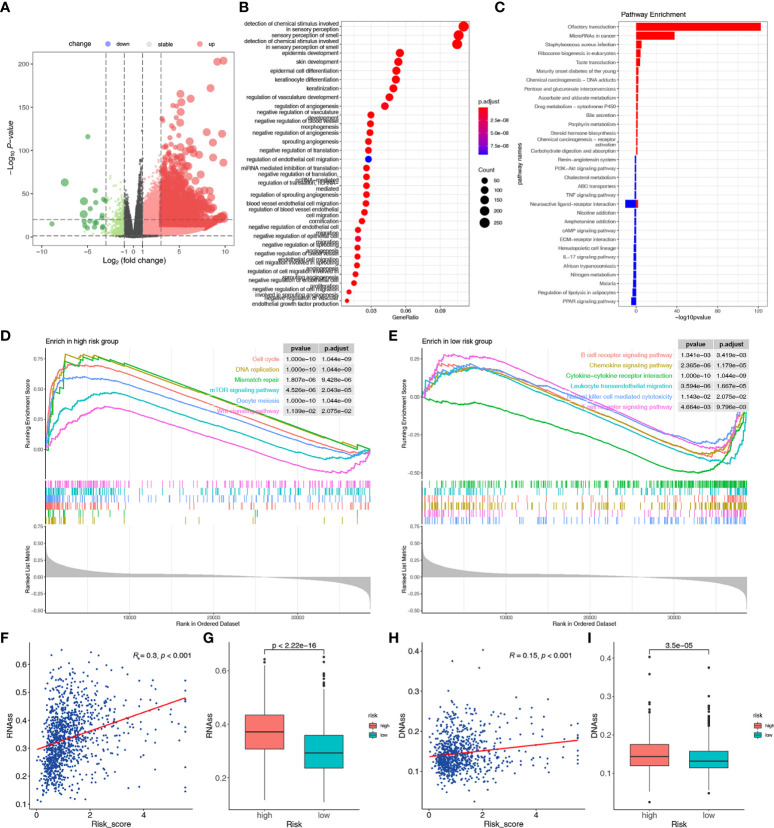
Functional enrichment, stemness, and m6A modification-related analyses between the two risk groups. **(A)** The volcano plot of the DEGs between the high-risk and low-risk groups. **(B)** The GO analysis of the DEGs. **(C)** The KEGG analysis of the DEGs. The pathways enriched in the high-risk **(D)** and low-risk **(E)** groups according to the GSEA. **(F)** The relationship between risk score and RNAss. **(G)** Differences in RNAss between the two risk groups. **(H)** The relationship between risk score and DNAss. **(I)** Differences in DNAss between the two risk groups.

To clarify the biological function characteristics of the two risk groups, we conducted GO, KEGG, and GSEA analyses. Using the R package “edgeR”, 20854 DEGs were identified between the two risk groups, with 20218 up-regulated and 636 down-regulated genes in the high-risk group (**Figure 6A**). GO analyses of the DEGs showed significant enrichment of immune-related biological processes, including regulation of angiogenesis (**Figure 6B**). Similarly, KEGG pathway analysis showed enrichment of immune-related pathways in the low-risk group, including the IL-17 signaling pathway and PPAR signaling pathway. In contrast, chemical carcinogenesis and microRNAs in cancer were activated in the high-risk group (**Figure 6C**). GSEA further verified that signatures related to cell cycle, mismatch repair, mTOR signaling, DNA replication, oocyte meiosis, and Wnt signaling pathways were significantly enriched in the high-risk group, indicating the proliferative status of high-risk patients (**Figure 6D**). The results also showed that immune-related pathways were enriched in the low-risk group, such as the T/B cell receptor signaling pathway, cytokine-cytokine receptor interaction, leukocyte transendothelial migration, natural killer cell-mediated cytotoxicity, and chemokine signaling pathway (**Figure 6E**). These results showed the different immune activity and proliferative status in the two risk groups, which might account for the different survival rates.

### Tumor stemness analyses

Stemness-related biomarkers in tumor cells were closely associated with drug resistance, cancer recurrence, and proliferation (62). We found that the risk score was positively correlated with RNAss and DNAss, and the two stemness-related biomarkers were higher in the high-risk group than in the low-risk score (p < 0.001) (**Figures 6F–I**). These results suggested that a high-risk score might indicate more active tumor-initiating cells.

### Different immune landscapes of two risk groups

**Figure 7:**
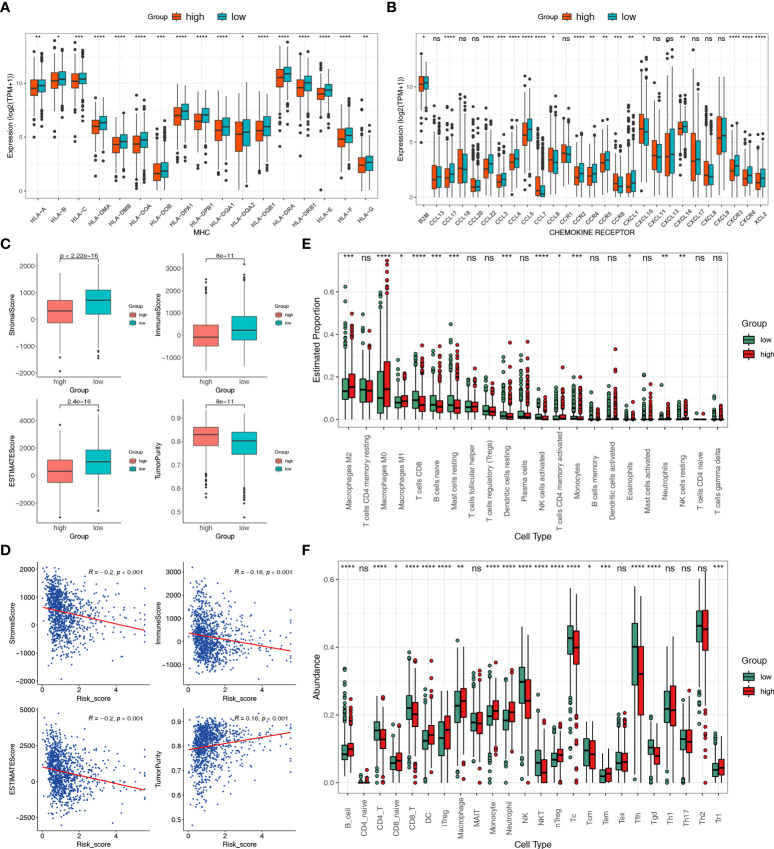
Different immune landscapes of the two risk groups. **(A)** Differences in MHC molecules between the two risk groups. **(B)** Differences in chemokines and receptors between the two risk groups. **(C)** Differences in tumor purity, immune, stromal, and estimated scores between the two risk groups. **(D)** Correlation between the tumor purity, immune, stromal, and estimated scores with the risk score. **(E)** Differences in infiltration fractions of 22 immune cell subsets between the two risk groups according to the CIBERSORTx. **(F)** Differences in infiltration fractions of 24 immune cell subsets between the two risk groups according to the ImmuCellAI database. Statistical significance at the level of ns>0.05, *≤0.05, **≤0.01, ***≤0.001 and ****≤0.0001.

Functional enrichment analysis revealed different degrees of immune function enrichment in the two risk groups. To investigate the characteristics of the tumor immune microenvironment (TIME) in the two risk groups, we estimated the expression of immunomodulators (54), immune checkpoint genes, and infiltration level of tumor-infiltrating immune cells. We found that expression of MHC-I constituents and MHC-II components were significantly elevated in the low-risk group (**Figure 7A**), indicating enhanced antigen presentation and processing capacity in low-risk patients. Key chemokines and their receptors included B2M, CCL17, CCL22, CCL3, CCL4, CCL5, CCR2, CCR4, CCR5, CXCL1, and CXCL16, CXCR3, CXCR6, and XCL2 were also significantly upregulated in the low-risk group (**Figure 7B**), suggesting that additional anti-tumor immune cells might be recruited in low-risk patients.

Furthermore, association analyses of immune components were performed, including ESTIMATE, Cibersortx, and ImmucellAI. The results of the ESTIMATE algorithm showed that the stromal, immune, and ESTIMATE scores were significantly higher in the low-risk group than in the high-risk group, while tumor purity was markedly increased in the high-risk group (**Figure 7C**). There was also a significant positive correlation between risk score and stromal, immune, and ESTIMATE scores, alongside a negative correlation with tumor purity (**Figure 7D**). High tumor purity is associated with cancer development and poor prognosis (63). CIBERSORTx can reveal the infiltration of immune cells in the TME. Moreover, M1 macrophages, CD8+ T cells, naïve B cells, resting mast cells, resting dendritic cells, activated natural killer (NK) cells, and monocytes were abundant in the low-risk group. In contrast, M2 macrophages, M0 macrophages, and resting NK cells were more predominant in the high-risk group (**Figure 7E**). As T cells have many subsets with specific functions, we assessed the abundance of infiltrated immune cells using the ssGSEA algorithm in the ImmuCellAI database (51). Several cell types, namely CD4+, CD8+, NK, NK T (NKT), Tc, Tcm, Tfh, and gamma delta T cells (Tgd) were markedly enriched in the low-risk group. In contrast, many immunosuppressive cell types were prevalent in the high-risk group, including B cells, naïve CD8, iTregs, macrophages, monocytes, neutrophils, dendritic cells (DC), natural regulatory T cells (nTregs), Tem, and type 1 regulatory T cells (Tr1) (**Figure 7F**). These results indicated that HLMRPM could predict TIME, and high-risk patients usually had lower immune infiltration and elevated immunosuppressive cells. These might partly explain the significant difference in prognosis between subgroups.

### Prediction of response to immunotherapy in BC patients

**Figure 8:**
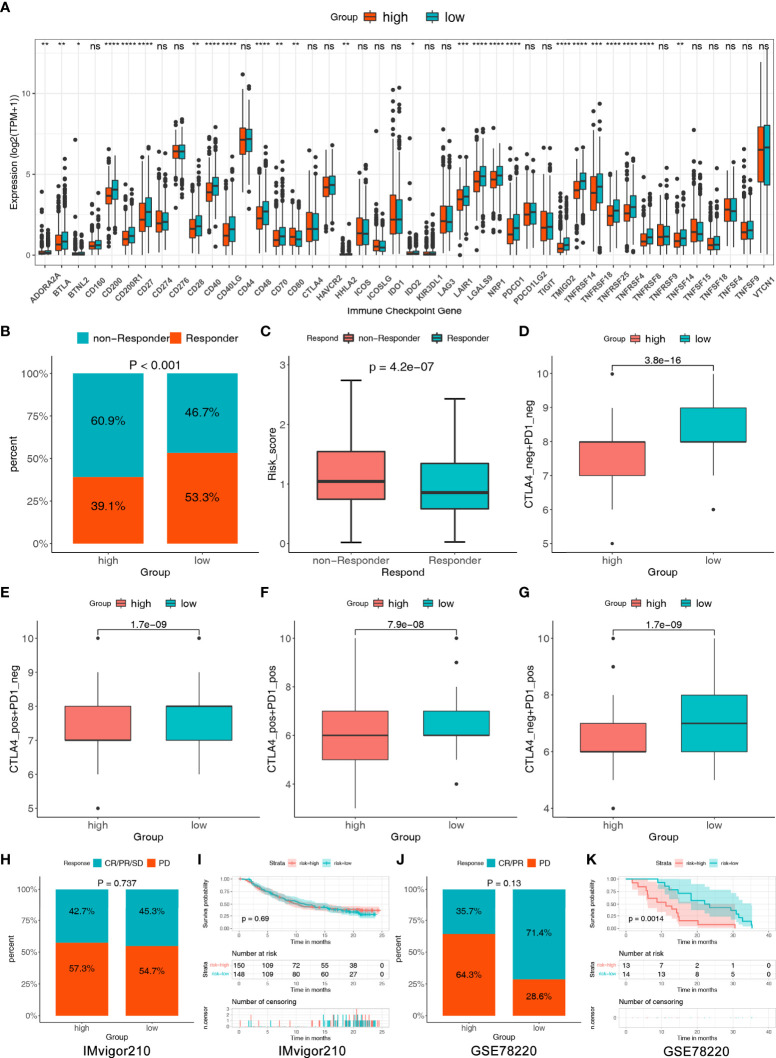
The assessment of immunotherapy response between the two risk groups. **(A)** Comparisons of the 44 ICGs in the two risk groups. **(B)** Comparisons of the proportions of non-responders and responders to ICIs between the two risk groups. **(C)** Differences in risk score between the responders and non-responders. **(D–G)** Differences in the IPS between the two risk groups stratified by CTLA4 and PD-1. **(H)** The proportion of patients with response to anti-PD-1/L1 immunotherapy in patients with high or low risk score in IMvigor210 cohort. **(I) **Survival analyses for patients with high or low risk score in IMvigor210 cohort. **(J)** The proportion of patients with response to anti-PD-1/L1 immunotherapy in patients with high or low risk score in GSE78220 cohort. **(K) **Survival analyses for patients with high or low risk score in GSE78220 cohort. Statistical significance at the level of ns>0.05, *≤0.05, **≤0.01, ***≤0.001 and ****≤0.0001.

Studies have shown that blocking immune checkpoint pathways could be a promising way to achieve anti-cancer immunity and high expression of ICGs related to a better response to ICIs (64). Therefore, we assessed the expression of 44 ICGs in the two risk groups. The results showed that nearly all ICGs were significantly higher in the low-risk group, such as BTLA, CD28, CD40, CD27 and PDCD1 (**Figure 8A**). We further evaluated the response of immunotherapy to TIDE and IPS. The results of TIDE showed that low-risk patients respond better to ICIs than high-risk patients (53.3% vs. 39.1%, *P* < 0.001) (**Figure 8B**). In addition, we found that the risk score was lower in responders than in non-responders, indicating a correlation between risk score and immunotherapy efficacy (**Figure 8C**). The susceptibility of patients to ICIs was further assessed using IPS. The results demonstrated that the low-risk group had higher IPS in any CTLA4 and PD-L1 stratification than the high-risk group, indicating that the relative probabilities of responding to ICIs in the low-risk group were higher than those in the high-risk group (**Figures 8D–G**).

To further test the capability of our model on immunotherapeutic benefit prediction, we utilized two common real-world immunotherapy cohorts (anti-PD-L1 in the IMvigor210 cohort and anti-PD-1 in the GSE78220 cohort). As shown in **Figures 8H–I**, in the IMvigor210 cohort, patients with a low-risk score showed a high proportion of response to anti-PD-L1, although there was no statistical difference (low versus high, 45.3 verse 42.7%, Chi-square test, p=0.737), and survival rate showed no difference in patients with high and low-risk groups. In the GSE78220 cohort, the frequency of CR/PR was also higher in the low-risk group (low versus high, 71.4 versus 35.7%, Chi-square test, p = 0.13); furthermore, the survival rate showed a significant difference in patients with high and low-risk groups (**Figures 8J–K**).

As a result, the two risk groups based on HLMRPM responded differently to immunotherapy, and patients with low risk might be sensitive to immunotherapy and attain more satisfactory clinical outcomes.

### HLMRPM predicts efficacy of chemotherapy response

**Figure 9:**
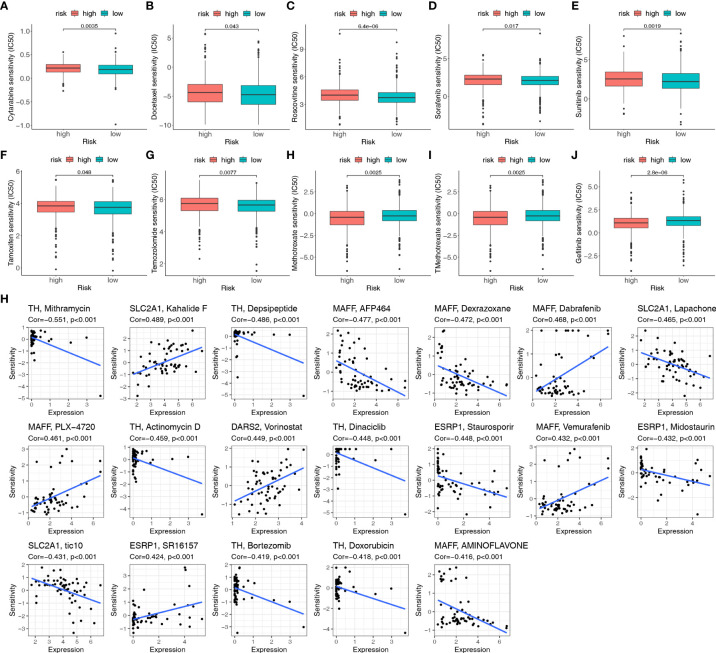
The sensitivity of chemotherapeutic agents and the prediction of potential drugs. **(A–J)** Comparison of the IC50 values of chemotherapy and targeted agents in the two risk groups, including cytarabine, docetaxel, sorafenib, temozolomide, tamoxifen, roscovitine, sunitinib, gefitinib, methotrexate, and TMethotrexate. **(H)** Sensitivity correlation analyses of the HLMRGs and potential drugs according to the CellMiner database.

To further enhance the clinical value of HLMRPM for treating BC, we predicted the efficacy of chemotherapy and potential agents for BC patients with “pRRophetic” and CellMiner database. We first calculated the IC50 for common chemotherapeutic agents against BC with the “pRRophetic” algorithm and compared the IC50 between the two risk groups. The IC50 value was the opposite of the sensitivity of the drugs. We found that low-risk patients were more sensitive to cytarabine, docetaxel, sorafenib, temozolomide, tamoxifen, roscovitine, and sunitinib than high-risk patients. Simultaneously, high-risk patients were more sensitive to gefitinib, methotrexate, and TMethotrexate (*P* < 0.05) (**Figures 9A–J**).

Moreover, 19 drugs targeting HLMRGs are available for treating BC according to CellMiner. TH was negatively associated with sensitivity to mithramycin, depsipeptide, actinomycin D, dinaciclib, bortezomib, and doxorubicin. MAFF was positively related to dabrafenib, PLX-4720, and vemurafenib, but negatively related to AFP464, dexrazoxane, and aminoflavone. ESRP1 was positively correlated with SR16157 and negatively correlated with staurosporine and midostaurin. SLC2A1 was positively correlated with kahalide F but negatively related to lapachone and tic10. The sensitivity to vorinostat was positively correlated with DARS2 (**Figure 9H**; **Table S4**). Based on these findings, the risk score can guide patients in receiving more appropriate drug treatment.

### Multi-omics validation of the nine HLMRGs

**Figure 10:**
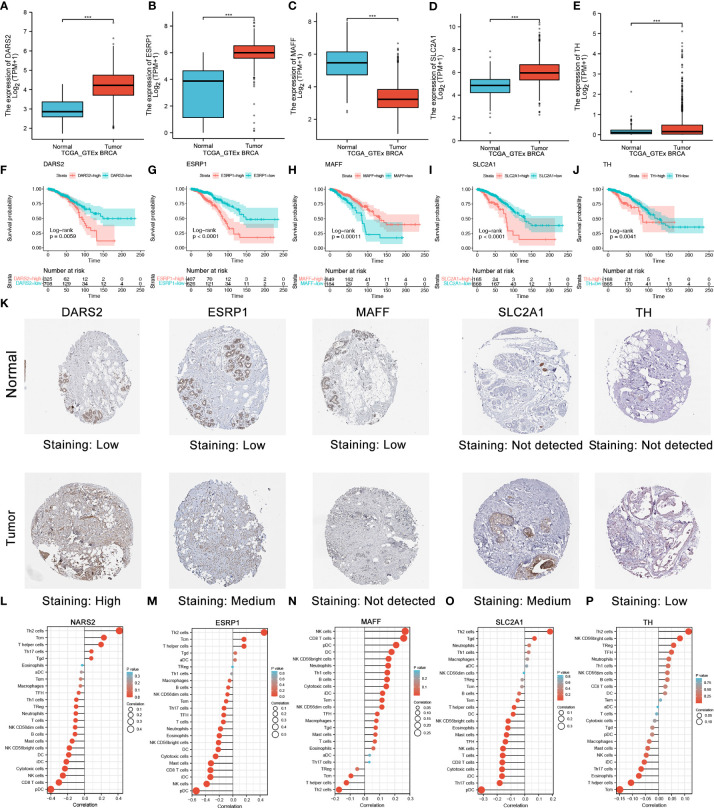
Validation of five selected HLMRGs. **(A–E)** Comparisons of the five HLMRGs between the BC and normal tissues combined with GTEx data. **(F–J)** Survival curve analysis of five HLMRGs based on TCGA. **(K)** Immunohistochemical staining for five HLMRGs in the normal breast tissue and BC. **(L–P)** Relationship between NARS2, ESRP1, MAFF, SLC2A1, and TH levels and immune cell infiltration based on ssGSEA. Statistical significance at the level of ***≤0.001.

To identify the role of the five HLMRGs in BC, we analyzed their mRNA expression, protein expression, function, and immunity. **Figures 10A–E** showed that, based on GTEx and TCGA databases, all five HLMRGs were differentially expressed between BC and normal samples. DARS2, ESRP1, SLC2A1, and TH were increased in BC, whereas MAFF was increased in normal tissues. Survival analysis indicated that high expression of DARS2, ESRP1, SLC2A1, and TH were related to poor prognosis, while high expression of MAFF was linked to better prognosis (**Figures 10F–J**).

We further verified the expression of the five HLRPGs using immunohistochemical images from the HPA database. We found that protein expressions of DARS2, ESRP1, SLC2A1, and TH were markedly high in BC tissues, whereas protein expression of MAFF was low in BC tissues (**Figure 10K**). We further summarized the immunohistochemistry staining characteristics of the five HLRPGs, and the results was coincident with above (**Figure S1**).

We further explored the associated functions of HLMRGs. We observed that DARS2, ESRP1, TH, and SLC2A1 might have an activation role in the apoptosis, cell cycle, DNA damage, and TSCmTOR signaling pathways while inhibiting the EMT, hormones AR and ER (**Figure S2**). Considering that HLMRPM was associated with TIME, we further explored the association between HLMRGs and the level of immune cell infiltration. The results showed that ESRP1 was most negatively correlated with DC and CD8+ cell infiltration. Other HLMRGs were also associated with the degree of immune cell infiltration (**Figures 10L-P**). These results demonstrated that the nine HLMRGs might have a critical role in the TIME and oncogenesis of BC, especially ESRP1.

## Discussion

Hypoxia and lactate metabolism are essential components of TME and are closely related to the occurrence and metastasis of cancer, drug resistance, immune infiltration, and inflammation (4, 5, 15–18, 27). In this study, we constructed an HMLRPM using rigorous bioinformatics and machine learning to predict prognosis, function, and therapy response in patients with BC.

Based on the HLMRPM-related risk score, all patients were classified into either high- or low-risk group. Multidimensional verification and evaluation of the model was conducted, resulting in the model being capable of independently and stably predicting BC prognoses. Gene enrichment analysis showed differing immune-related pathway activities between the two groups. The low-risk group was significantly enriched with gene sets reflecting positive immune function, such as cytokine-cytokine receptor interaction, T/B cell receptor signaling pathway, and chemokine signaling pathway, revealing that increased immune activity might be related to the better prognosis of low-risk patients.

Further analyses showed significant differences in immune cell infiltration between the two risk groups. ESTIMATE is an algorithm used to estimate immune cells, stromal cells, and tumor purity (52). We found that the low-risk group had higher stromal, immune, and ESTIMATE scores than the low-risk group. Previous studies have demonstrated that immune and stromal cells are prognostic factors for tumors (65, 66). TME is composed of tissue-resident cells, recruits tumor-infiltrating immune cells, and plays a crucial role in tumor progression and metastasis (67, 68). Cibersortx and ImmuCellAI were used to assess the proportion of immune cells. The results showed that the low-risk group had many anti-tumor cells, such as CD8+, CD4+, and activated NK cells. In contrast, many immunosuppressive cell types such as macrophages M0 and M2 are prevalent in high-risk patients. Infiltration of immune cells is a critical determinant of tumor prognosis and progression (69, 70). In BC, tumor-infiltrating lymphocytes (TILs) modulate the response to chemotherapy and improve clinical outcomes. Macrophage infiltration can result in angiogenesis, enhanced tumor cell mobility, and poor survival in BC (71). Tregs may induce immune tolerance and facilitate immune escape and tumor metastasis (72, 73). Our findings are confirmed by these studies, namely, that the prognosis of high-risk groups with increased immunosuppressive cell infiltration is poor.

ICIs are critical in treating multiple cancer types (74). However, previous research has shown that only 12.6% of cancer patients respond to ICIs (75). Developing predictive biomarkers for ICI treatment has always been important for screening treatment populations to achieve precise treatment. Many predictive biomarkers of ICI therapy have been developed, such as PD-L1 expression and CD8 infiltration (76, 77). Our study used existing biomarkers and related databases to evaluate the treatment response to ICIs in the two risk groups. We found that the expression of most immune checkpoints was significantly increased in the low-risk group, indicating that low-risk patients could better respond to ICIs (78). Furthermore, TIDE and IPS consistently showed that low-risk patients responded better to immunotherapy treatment. These results showed that low-risk patients had more anti-tumor immune infiltrating cells and a better immunotherapy response. The estimated results from two immunotherapy cohorts indicated the potential association between risk score and the curative effect of immunotherapy. This might impact the survival and prognosis of BC patients, and the model might act as a biomarker for ICI therapy in BC.

The essential roles of these five HLRPGs have been studied in various cancer types, including BC. Epithelial splicing regulatory protein 1 (ESRP1) may be a new drug resistance biomarker and therapeutic target for patients with small cell lung cancer [SCLC] (79). ESRP1 can increase intracellular GSH levels and the metastatic lung potential of BC (80). ESRP1 is also associated with epithelial-mesenchymal transition and chemoresistance in multiple cancers (81, 82). DARS2 has been identified as a hepatocellular carcinoma (HCC) oncogene that promotes HCC cell cycle progression and inhibits HCC cell apoptosis (83). In lung adenocarcinoma cells, DARS2 is involved in proliferation, invasion, and apoptosis and shows promise as a therapeutic target (84). MAFF could promote tumor invasion and metastasis through IL11 and STAT3 signaling (85). SLC2A1 overexpression correlates with the suppression of CD8+ T cells and B cells in gastric cancer (86). In LUAD and HCC, SLC2A1 plays a significant prognostic role and is associated with tumor immunity (87, 88). TH expression in neuroblastoma predicts poor survival and is an independent prognostic factor (89). Our study found that ESRP1 was the most potent biomarker among the five HLMRGs. It is significantly upregulated in BC and is related to epithelial-mesenchymal transition. Moreover, it was negatively correlated with anti-tumor immune infiltration cells.

However, our study has several limitations. First, our research is based on analyzing existing databases; therefore, further validation of HLMRPM in a large cohort is needed. Moreover, an in-depth characterization of the mechanisms of the discovered HLMRGs needs to be conducted through cell and animal experiments.

## Conclusions

In summary, our study combined hypoxia-lactate metabolism-related genes to construct a prognostic signature for BC using machine learning and bioinformatics. The HLRPM could identify high-risk populations, predict immune infiltration, immunotherapy, and chemotherapy sensitivity. Validation of HLMRGs demonstrate the potential biomarker value of HLMRGs, which could assist in selecting the appropriate treatment population and improving the prognosis of patients with BC.

## Data availability statement

Publicly available datasets were analyzed in this study. This data can be found here: https://portal.gdc.cancer.gov/repository, http://www.ncbi.nlm.nih.gov/geo/, https://www.proteinatlas.org/, and https://xenabrowser.net/datapages/.

## Author contributions

JL, SZ and ZZ conceptualized and designed the study. FW, SS, HQ, WY, CL, WL, and CF prepared the dataset. JL, ML, FW, and HQ performed the data analyses and prepared the figures and tables. JL, HW, XC, YC, and YuZ interpreted the data. JL, XL, and ZZ wrote and revised the manuscript. SZ and YiZ supervised the study. All authors contributed to the article and approved the submitted version.

## Funding

This work is supported by National Natural Science Foundation of China (No. 82103212).

## Acknowledgments

We sincerely thank the contributors in the TCGA, GTEx, GEO, and HPA projects and the Editage (www.editage.cn) for English language editing.

## Conflict of interest

The authors declare that the research was conducted in the absence of any commercial or financial relationships that could be construed as a potential conflict of interest.

## Publisher’s note

All claims expressed in this article are solely those of the authors and do not necessarily represent those of their affiliated organizations, or those of the publisher, the editors and the reviewers. Any product that may be evaluated in this article, or claim that may be made by its manufacturer, is not guaranteed or endorsed by the publisher.

## Supplementary material

Supplementary Figure 1 The immunohistochemistry staining characteristics of the five HLRPGsfrom the HPA database.

Supplementary Figure 2 The heatmap showed the correlation between five HLMRGs expressionsand the critical cancer signaling pathways.

Supplementary Table 2 A total of 284 lactate-related genes and 493-hypoxia related genes usedin this study.

## References

[1] Siegel RL Miller KD Fuchs HE Jemal A. Cancer statistics, 2022. CA Cancer J Clin (2022) 72(1):7–33. doi: 10.3322/caac.21708 35020204

[2] Ali HR Glont SE Blows FM Provenzano E Dawson SJ Liu B. PD-L1 protein expression in breast cancer is rare, enriched in basal-like tumours and associated with infiltrating lymphocytes. Ann Oncol (2015) 26(7):1488–93. doi: 10.1093/annonc/mdv192 25897014

[3] Darby S McGale P Correa C Taylor C Arriagada R Clarke M. Effect of radiotherapy after breast-conserving surgery on 10-year recurrence and 15-year breast cancer death: Meta-analysis of individual patient data for 10,801 women in 17 randomised trials. Lancet (2011) 378(9804):1707–16. doi: 10.1016/S0140-6736(11)61629-2 PMC3254252 22019144

[4] Shao C Yang F Miao S Liu W Wang C Shu Y. Role of hypoxia-induced exosomes in tumor biology. Mol Cancer (2018) 17(1):120. doi: 10.1186/s12943-018-0869-y 30098600 PMC6087002

[5] Vaupel P Mayer A Höckel M. Tumor hypoxia and malignant progression. Methods Enzymol (2004) 381:335–54. doi: 10.1016/S0076-6879(04)81023-1 15063685

[6] Harris AL. Hypoxia–a key regulatory factor in tumour growth. Nat Rev Cancer (2002) 2(1):38–47. doi: 10.1038/nrc704 11902584

[7] Jing X Yang F Shao C Wei K Xie M Shen H. Role of hypoxia in cancer therapy by regulating the tumor microenvironment. Mol Cancer (2019) 18(1):157. doi: 10.1186/s12943-019-1089-9 31711497 PMC6844052

[8] Vaupel P Mayer A. Hypoxia in cancer: significance and impact on clinical outcome. Cancer Metastasis Rev (2007) 26(2):225–39. doi: 10.1007/s10555-007-9055-1 17440684

[9] Klein TJ Glazer PM. The tumor microenvironment and DNA repair. Semin Radiat Oncol (2010) 20(4):282–7. doi: 10.1016/j.semradonc.2010.05.006 PMC2948843 20832021

[10] Graeber TG Osmanian C Jacks T Housman DE Koch CJ Lowe SW. Hypoxia-mediated selection of cells with diminished apoptotic potential in solid tumours. Nature (1996) 379(6560):88–91. doi: 10.1038/379088a0 8538748

[11] Suda T Takubo K Semenza GL. Metabolic regulation of hematopoietic stem cells in the hypoxic niche. Cell Stem Cell (2011) 9(4):298–310. doi: 10.1016/j.stem.2011.09.010 21982230

[12] Movahedi K Laoui D Gysemans C Baeten M Stangé G Van den Bossche J. Different tumor microenvironments contain functionally distinct subsets of macrophages derived from Ly6C(high) monocytes. Cancer Res (2010) 70(14):5728–39. doi: 10.1158/0008-5472.CAN-09-4672 20570887

[13] To KKW Sedelnikova OA Samons M Bonner WM Huang LE. The phosphorylation status of PAS-b distinguishes HIF-1alpha from HIF-2alpha in NBS1 repression. EMBO J (2006) 25(20):4784–94. doi: 10.1038/sj.emboj.7601369 PMC1618093 17024177

[14] Semenza GL. Hypoxia-inducible factors: mediators of cancer progression and targets for cancer therapy. Trends Pharmacol Sci (2012) 33(4):207–14. doi: 10.1016/j.tips.2012.01.005 PMC3437546 22398146

[15] Semenza GL. HIF-1 mediates metabolic responses to intratumoral hypoxia and oncogenic mutations. J Clin Invest (2013) 123(9):3664–71. doi: 10.1172/JCI67230 PMC3754249 23999440

[16] Doherty JR Cleveland JL. Targeting lactate metabolism for cancer therapeutics. J Clin Invest (2013) 123(9):3685–92. doi: 10.1172/JCI69741 PMC3754272 23999443

[17] Hayes C Donohoe CL Davern M Donlon NE. The oncogenic and clinical implications of lactate induced immunosuppression in the tumour microenvironment. Cancer Lett (2021) 500:75–86. doi: 10.1016/j.canlet.2020.12.021 33347908

[18] Hillis AL Toker A. Lactate lights up PI3K inhibitor resistance in breast cancer. Cancer Cell (2020) 38(4):441–3. doi: 10.1016/j.ccell.2020.09.011 33049205

[19] Osthus RC Shim H Kim S Li Q Reddy R Mukherjee M. Deregulation of glucose transporter 1 and glycolytic gene expression by c-myc. J Biol Chem (2000) 275(29):21797–800. doi: 10.1074/jbc.C000023200 10823814

[20] Jin Z Lu Y Wu X Pan T Yu Z Hou J. The cross-talk between tumor cells and activated fibroblasts mediated by lactate/BDNF/TrkB signaling promotes acquired resistance to anlotinib in human gastric cancer. Redox Biol (2021) 46:102076. doi: 10.1016/j.redox.2021.102076 34315112 PMC8326414

[21] Brown TP Bhattacharjee P Ramachandran S Sivaprakasam S Ristic B Sikder MOF. The lactate receptor GPR81 promotes breast cancer growth *via* a paracrine mechanism involving antigen-presenting cells in the tumor microenvironment. Oncogene (2020) 39(16):3292–304. doi: 10.1038/s41388-020-1216-5 32071396

[22] Rossi V Govoni M Farabegoli F Di Stefano G. Lactate is a potential promoter of tamoxifen resistance in MCF7 cells. Biochim Biophys Acta Gen Subj (2022) 1866(9):130185. doi: 10.1016/j.bbagen.2022.130185 35661802

[23] Martinez-Outschoorn UE Prisco M Ertel A Tsirigos A Lin Z Pavlides S. Ketones and lactate increase cancer cell “stemness,” driving recurrence, metastasis and poor clinical outcome in breast cancer: Achieving personalized medicine *via* metabolo-genomics. Cell Cycle (2011) 10(8):1271–86. doi: 10.4161/cc.10.8.15330 PMC3117136 21512313

[24] Facciabene A Peng X Hagemann IS Balint K Barchetti A Wang L-P. Tumour hypoxia promotes tolerance and angiogenesis *via* CCL28 and t(reg) cells. Nature (2011) 475(7355):226–30. doi: 10.1038/nature10169 21753853

[25] Sitkovsky M Lukashev D. Regulation of immune cells by local-tissue oxygen tension: HIF1 alpha and adenosine receptors. Nat Rev Immunol (2005) 5(9):712–21. doi: 10.1038/nri1685 16110315

[26] Peng M Yin N Chhangawala S Xu K Leslie CS Li MO. Aerobic glycolysis promotes T helper 1 cell differentiation through an epigenetic mechanism. Science (2016) 354(6311):481–4. doi: 10.1126/science.aaf6284 PMC5539971 27708054

[27] Ivashkiv LB. The hypoxia-lactate axis tempers inflammation. Nat Rev Immunol (2020) 20(2):85–6. doi: 10.1038/s41577-019-0259-8 PMC7021227 31819164

[28] Corbet C Feron O. Tumour acidosis: from the passenger to the driver’s seat. Nat Rev Cancer (2017) 17(10):577–93. doi: 10.1038/nrc.2017.77 28912578

[29] Erra Díaz F Dantas E Geffner J. Unravelling the interplay between extracellular acidosis and immune cells. Mediators Inflamm (2018) 2018:1218297. doi: 10.1155/2018/1218297 30692870 PMC6332927

[30] Nakagawa Y Negishi Y Shimizu M Takahashi M Ichikawa M Takahashi H. Effects of extracellular pH and hypoxia on the function and development of antigen-specific cytotoxic T lymphocytes. Immunol Lett (2015) 167(2):72–86. doi: 10.1016/j.imlet.2015.07.003 26209187

[31] Barsoum IB Smallwood CA Siemens DR Graham CH. A mechanism of hypoxia-mediated escape from adaptive immunity in cancer cells. Cancer Res (2014) 74(3):665–74. doi: 10.1158/0008-5472.CAN-13-0992 24336068

[32] Noman MZ Desantis G Janji B Hasmim M Karray S Dessen P. PD-L1 is a novel direct target of HIF-1α, and its blockade under hypoxia enhanced MDSC-mediated T cell activation. J Exp Med (2014) 211(5):781–90. doi: 10.1084/jem.20131916 PMC4010891 24778419

[33] Kumagai S Koyama S Itahashi K Tanegashima T Lin Y-T Togashi Y. Lactic acid promotes PD-1 expression in regulatory T cells in highly glycolytic tumor microenvironments. Cancer Cell (2022) 40(2):201–18. doi: 10.1016/j.ccell.2022.01.001 35090594

[34] Xie S Ding B Wang S Zhang X Yan W Xia Q. Construction of hypoxia-immune-related prognostic model and targeted therapeutic strategies for cervical cancer. Int Immunol (2022) 7:379–94. doi: 10.1093/intimm/dxac017 35561666

[35] Zhu G-L Yang K-B Xu C Feng R-J Li W-F Ma J. Development of a prediction model for radiotherapy response among patients with head and neck squamous cell carcinoma based on the tumor immune microenvironment and hypoxia signature. Cancer Med (2022). doi: 10.1002/cam4.4791 PMC9741991 35505641

[36] Yan D Cai S Bai L Du Z Li H Sun P. Integration of immune and hypoxia gene signatures improves the prediction of radiosensitivity in breast cancer. Am J Cancer Res (2022) 12(3):1222–40. https://pubmed.ncbi.nlm.nih.gov/35411250/ PMC8984882 35411250

[37] Xie Y Zhang J Li M Zhang Y Li Q Zheng Y. Identification of lactate-related gene signature for prediction of progression and immunotherapeutic response in skin cutaneous melanoma. Front Oncol (2022) 12:818868. doi: 10.3389/fonc.2022.818868 35265521 PMC8898832

[38] Sun Z Tao W Guo X Jing C Zhang M Wang Z. Construction of a lactate-related prognostic signature for predicting prognosis, tumor microenvironment, and immune response in kidney renal clear cell carcinoma. Front Immunol (2022) 13:818984. doi: 10.3389/fimmu.2022.818984 35250999 PMC8892380

[39] Yang L Tan P Sun H Zeng Z Pan Y. Integrative dissection of novel lactate metabolism-related signature in the tumor immune microenvironment and prognostic prediction in breast cancer. Front Oncol (2022) 12:874731. doi: 10.3389/fonc.2022.874731 35574387 PMC9094627

[40] Sun X Luo H Han C Zhang Y Yan C. Identification of a hypoxia-related molecular classification and hypoxic tumor microenvironment signature for predicting the prognosis of patients with triple-negative breast cancer. Front Oncol (2021) 11:700062. doi: 10.3389/fonc.2021.700062 34490098 PMC8416750

[41] Zhao Y Liu L Zhao J Du X Yu Q Wu J. Construction and verification of a hypoxia-related 4-lncRNA model for prediction of breast cancer. Int J Gen Med (2021) 14:4605–17. doi: 10.2147/IJGM.S322007 PMC8380141 34429643

[42] Mariathasan S Turley SJ Nickles D Castiglioni A Yuen K Wang Y. TGFβ attenuates tumour response to PD-L1 blockade by contributing to exclusion of T cells. Nature (2018) 554(7693):544–8. doi: 10.1038/nature25501 PMC6028240 29443960

[43] Hugo W Zaretsky JM Sun L Song C Moreno BH Hu-Lieskovan S. Genomic and transcriptomic features of response to anti-PD-1 therapy in metastatic melanoma. Cell (2016) 165(1):35–44. doi: 10.1016/j.cell.2016.02.065 26997480 PMC4808437

[44] Liberzon A Birger C Thorvaldsdóttir H Ghandi M Mesirov JP Tamayo P. The molecular signatures database (MSigDB) hallmark gene set collection. Cell Syst (2015) 1(6):417–25. doi: 10.1016/j.cels.2015.12.004 PMC4707969 26771021

[45] Ishwaran H Kogalur UB Blackstone EH Lauer MS. Random survival forests. Ann Appl Statistics (2008) 2(3):841–60, 20. doi: 10.1214/08-AOAS169

[46] Taylor JMG. Random survival forests. J Thorac Oncol (2011) 6(12):1974–5. doi: 10.1097/JTO.0b013e318233d835 22088987

[47] Chen X Ishwaran H. Random forests for genomic data analysis. Genomics (2012) 99(6):323–9. doi: 10.1016/j.ygeno.2012.04.003 PMC3387489 22546560

[48] Yu G Wang L-G Han Y He Q-Y. clusterProfiler: an r package for comparing biological themes among gene clusters. OMICS (2012) 16(5):284–7. doi: 10.1089/omi.2011.0118 PMC3339379 22455463

[49] Subramanian A Kuehn H Gould J Tamayo P Mesirov JP. GSEA-p: A desktop application for gene set enrichment analysis. Bioinformatics (2007) 23(23):3251–3. doi: 10.1093/bioinformatics/btm369 17644558

[50] Malta TM Sokolov A Gentles AJ Burzykowski T Poisson L Weinstein JN. Machine learning identifies stemness features associated with oncogenic dedifferentiation. Cell (2018) 173(2):338–54. https://pubmed.ncbi.nlm.nih.gov/29625051/ 10.1016/j.cell.2018.03.034 PMC5902191 29625051

[51] Miao Y-R Zhang Q Lei Q Luo M Xie G-Y Wang H. ImmuCellAI: A unique method for comprehensive T-cell subsets abundance prediction and its application in cancer immunotherapy. Adv Sci (Weinh) (2020) 7(7):1902880. doi: 10.1002/advs.201902880 32274301 PMC7141005

[52] Yoshihara K Shahmoradgoli M Martínez E Vegesna R Kim H Torres-Garcia W. Inferring tumour purity and stromal and immune cell admixture from expression data. Nat Commun (2013) 4:2612. doi: 10.1038/ncomms3612 24113773 PMC3826632

[53] Newman AM Liu CL Green MR Gentles AJ Feng W Xu Y. Robust enumeration of cell subsets from tissue expression profiles. Nat Methods (2015) 12(5):453–7. doi: 10.1038/nmeth.3337 PMC4739640 25822800

[54] Charoentong P Finotello F Angelova M Mayer C Efremova M Rieder D. Pan-cancer immunogenomic analyses reveal genotype-immunophenotype relationships and predictors of response to checkpoint blockade. Cell Rep (2017) 18(1):248–62. doi: 10.1016/j.celrep.2016.12.019 28052254

[55] Fu J Li K Zhang W Wan C Zhang J Jiang P. Large-Scale public data reuse to model immunotherapy response and resistance. Genome Med (2020) 12(1):21. doi: 10.1186/s13073-020-0721-z 32102694 PMC7045518

[56] Jiang P Gu S Pan D Fu J Sahu A Hu X. Signatures of T cell dysfunction and exclusion predict cancer immunotherapy response. Nat Med (2018) 24(10):1550–8. doi: 10.1038/s41591-018-0136-1 PMC6487502 30127393

[57] Geeleher P Cox NJ Huang RS. Clinical drug response can be predicted using baseline gene expression levels and *in vitro* drug sensitivity in cell lines. Genome Biol (2014) 15(3):R47. doi: 10.1186/gb-2014-15-3-r47 24580837 PMC4054092

[58] Yang W Soares J Greninger P Edelman EJ Lightfoot H Forbes S. Genomics of drug sensitivity in cancer (GDSC): A resource for therapeutic biomarker discovery in cancer cells. Nucleic Acids Res (2013) 41(Database issue):D955–D61. doi: 10.1093/nar/gks1111 PMC3531057 23180760

[59] Shankavaram UT Reinhold WC Nishizuka S Major S Morita D Chary KK. Transcript and protein expression profiles of the NCI-60 cancer cell panel: an integromic microarray study. Mol Cancer Ther (2007) 6(3):820–32. doi: 10.1158/1535-7163.MCT-06-0650 17339364

[60] Shankavaram UT Varma S Kane D Sunshine M Chary KK Reinhold WC. CellMiner: A relational database and query tool for the NCI-60 cancer cell lines. BMC Genomics (2009) 10:277. doi: 10.1186/1471-2164-10-277 19549304 PMC2709662

[61] Vickers AJ Elkin EB. Decision curve analysis: a novel method for evaluating prediction models. Med Decis Making (2006) 26(6):565–74. doi: 10.1177/0272989X06295361 PMC2577036 17099194

[62] Luo Q Vögeli T-A. A methylation-based reclassification of bladder cancer based on immune cell genes. Cancers (Basel) (2020) 12(10):3054. doi: 10.3390/cancers12103054 PMC7593922 33092083

[63] Deng X Pan Y Yang M Liu Y Li J. PLOD3 is associated with immune cell infiltration and genomic instability in colon adenocarcinoma. BioMed Res Int (2021) 2021:4714526. doi: 10.1155/2021/4714526 34239923 PMC8235962

[64] Li B Chan HL Chen P. Immune checkpoint inhibitors: Basics and challenges. Curr Med Chem (2019) 26(17):3009–25. doi: 10.2174/0929867324666170804143706 28782469

[65] Mahajan UM Langhoff E Goni E Costello E Greenhalf W Halloran C. Immune cell and stromal signature associated with progression-free survival of patients with resected pancreatic ductal adenocarcinoma. Gastroenterology (2018) 155(5):1625–39. doi: 10.1053/j.gastro.2018.08.009 30092175

[66] Efstathiou JA Mouw KW Gibb EA Liu Y Wu C-L Drumm MR. Impact of immune and stromal infiltration on outcomes following bladder-sparing trimodality therapy for muscle-invasive bladder cancer. Eur Urol (2019) 76(1):59–68. doi: 10.1016/j.eururo.2019.01.011 30712971 PMC6571058

[67] Quail DF Joyce JA. Microenvironmental regulation of tumor progression and metastasis. Nat Med (2013) 19(11):1423–37. doi: 10.1038/nm.3394 PMC3954707 24202395

[68] Cassetta L Pollard JW. Targeting macrophages: therapeutic approaches in cancer. Nat Rev Drug Discovery (2018) 17(12):887–904. doi: 10.1038/nrd.2018.169 30361552

[69] Zhou R Zhang J Zeng D Sun H Rong X Shi M. Immune cell infiltration as a biomarker for the diagnosis and prognosis of stage I-III colon cancer. Cancer Immunol Immunother (2019) 68(3):433–42. doi: 10.1007/s00262-018-2289-7 PMC6426802 30564892

[70] Zuo S Wei M Wang S Dong J Wei J. Pan-cancer analysis of immune cell infiltration identifies a prognostic immune-cell characteristic score (ICCS) in lung adenocarcinoma. Front Immunol (2020) 11:1218. doi: 10.3389/fimmu.2020.01218 32714316 PMC7344231

[71] Kitamura T Qian B-Z Pollard JW. Immune cell promotion of metastasis. Nat Rev Immunol (2015) 15(2):73–86. doi: 10.1038/nri3789 25614318 PMC4470277

[72] Bauer CA Kim EY Marangoni F Carrizosa E Claudio NM Mempel TR. Dynamic treg interactions with intratumoral APCs promote local CTL dysfunction. J Clin Invest (2014) 124(6):2425–40. doi: 10.1172/JCI66375 PMC4089459 24812664

[73] Yang P Li Q-J Feng Y Zhang Y Markowitz GJ Ning S. TGF-β-miR-34a-CCL22 signaling-induced treg cell recruitment promotes venous metastases of HBV-positive hepatocellular carcinoma. Cancer Cell (2012) 22(3):291–303. doi: 10.1016/j.ccr.2012.07.023 22975373 PMC3443566

[74] Vaddepally RK Kharel P Pandey R Garje R Chandra AB. Review of indications of FDA-approved immune checkpoint inhibitors per NCCN guidelines with the level of evidence. Cancers (Basel) (2020) 12(3):738. doi: 10.3390/cancers12030738 PMC7140028 32245016

[75] Haslam A Prasad V. Estimation of the percentage of US patients with cancer who are eligible for and respond to checkpoint inhibitor immunotherapy drugs. JAMA Netw Open (2019) 2(5):e192535. doi: 10.1001/jamanetworkopen.2019.2535 31050774 PMC6503493

[76] Gibney GT Weiner LM Atkins MB. Predictive biomarkers for checkpoint inhibitor-based immunotherapy. Lancet Oncol (2016) 17(12):e542–e51. doi: 10.1016/S1470-2045(16)30406-5 PMC5702534 27924752

[77] McGranahan N Furness AJS Rosenthal R Ramskov S Lyngaa R Saini SK. Clonal neoantigens elicit T cell immunoreactivity and sensitivity to immune checkpoint blockade. Science (2016) 351(6280):1463–9. doi: 10.1126/science.aaf1490 PMC4984254 26940869

[78] Stanton SE Disis ML. Clinical significance of tumor-infiltrating lymphocytes in breast cancer. J Immunother Cancer (2016) 4:59. doi: 10.1186/s40425-016-0165-6 27777769 PMC5067916

[79] Zheng M Niu Y Bu J Liang S Zhang Z Liu J. ESRP1 regulates alternative splicing of CARM1 to sensitize small cell lung cancer cells to chemotherapy by inhibiting TGF-β/Smad signaling. Aging (Albany NY) (2021) 13(3):3554–72. doi: 10.18632/aging.202295 PMC7906186 33495408

[80] Yae T Tsuchihashi K Ishimoto T Motohara T Yoshikawa M Yoshida GJ. Alternative splicing of CD44 mRNA by ESRP1 enhances lung colonization of metastatic cancer cell. Nat Commun (2012) 3:883. doi: 10.1038/ncomms1892 22673910

[81] Yu S Wang M Zhang H Guo X Qin R. Circ_0092367 inhibits EMT and gemcitabine resistance in pancreatic cancer *via* regulating the miR-1206/ESRP1 axis. Genes (Basel) (2021) 12(11):1701. doi: 10.3390/genes12111701 34828307 PMC8622583

[82] Vadlamudi Y Dey DK Kang SC. Emerging multi-cancer regulatory role of ESRP1: Orchestration of alternative splicing to control EMT. Curr Cancer Drug Targets (2020) 20(9):654–65. doi: 10.2174/1568009620666200621153831 32564755

[83] Qin X Li C Guo T Chen J Wang H-T Wang Y-T. Upregulation of DARS2 by HBV promotes hepatocarcinogenesis through the miR-30e-5p/MAPK/NFAT5 pathway. J Exp Clin Cancer Res (2017) 36(1):148. doi: 10.1186/s13046-017-0618-x 29052520 PMC5649064

[84] Jiang Y You J Wu C Kang Y Chen F Chen L. High expression of DARS2 indicates poor prognosis in lung adenocarcinoma. J Clin Lab Anal (2022):e24691. doi: 10.1002/jcla.24691 36085578 PMC9550967

[85] Moon EJ Mello SS Li CG Chi J-T Thakkar K Kirkland JG. The HIF target MAFF promotes tumor invasion and metastasis through IL11 and STAT3 signaling. Nat Commun (2021) 12(1):4308. doi: 10.1038/s41467-021-24631-6 34262028 PMC8280233

[86] Min K-W Kim D-H Son BK Moon KM Kim SM Intazur Rahaman M. High SLC2A1 expression associated with suppressing CD8 T cells and b cells promoted cancer survival in gastric cancer. PloS One (2021) 16(3):e0245075. doi: 10.1371/journal.pone.0245075 33735188 PMC7971512

[87] Wang Y Wen H Sun D. Plays a significant prognostic role in lung adenocarcinoma and is associated with tumor immunity based on bioinformatics analysis. Ann Transl Med (2022) 10(9):519. doi: 10.1080/07853890.2021.2016942 35928739 PMC9347052

[88] Peng Q Hao L-Y Guo Y-L Zhang Z-Q Ji J-M Xue Y. Solute carrier family 2 members 1 and 2 as prognostic biomarkers in hepatocellular carcinoma associated with immune infiltration. World J Clin Cases (2022) 10(13):3989–4019. doi: 10.12998/wjcc.v10.i13.3989 35665115 PMC9131213

[89] Lee NH Son MH Choi YB Yi E Lee JW Yoo KH. Clinical significance of tyrosine hydroxylase mRNA transcripts in peripheral blood at diagnosis in patients with neuroblastoma. Cancer Res Treat (2016) 48(4):1399–407. doi: 10.4143/crt.2015.481 PMC5080821 27034145

