# Supplementary figures and images for: A novel hypoxia- and lactate metabolism-related signature to predict prognosis and immunotherapy responses for breast cancer by integrating machine learning and bioinformatic analyses
Source: Front Immunol. 2022 Oct 7;13:998140. doi: 10.3389/fimmu.2022.998140 (PMC9585224; doi:10.3389/fimmu.2022.998140)

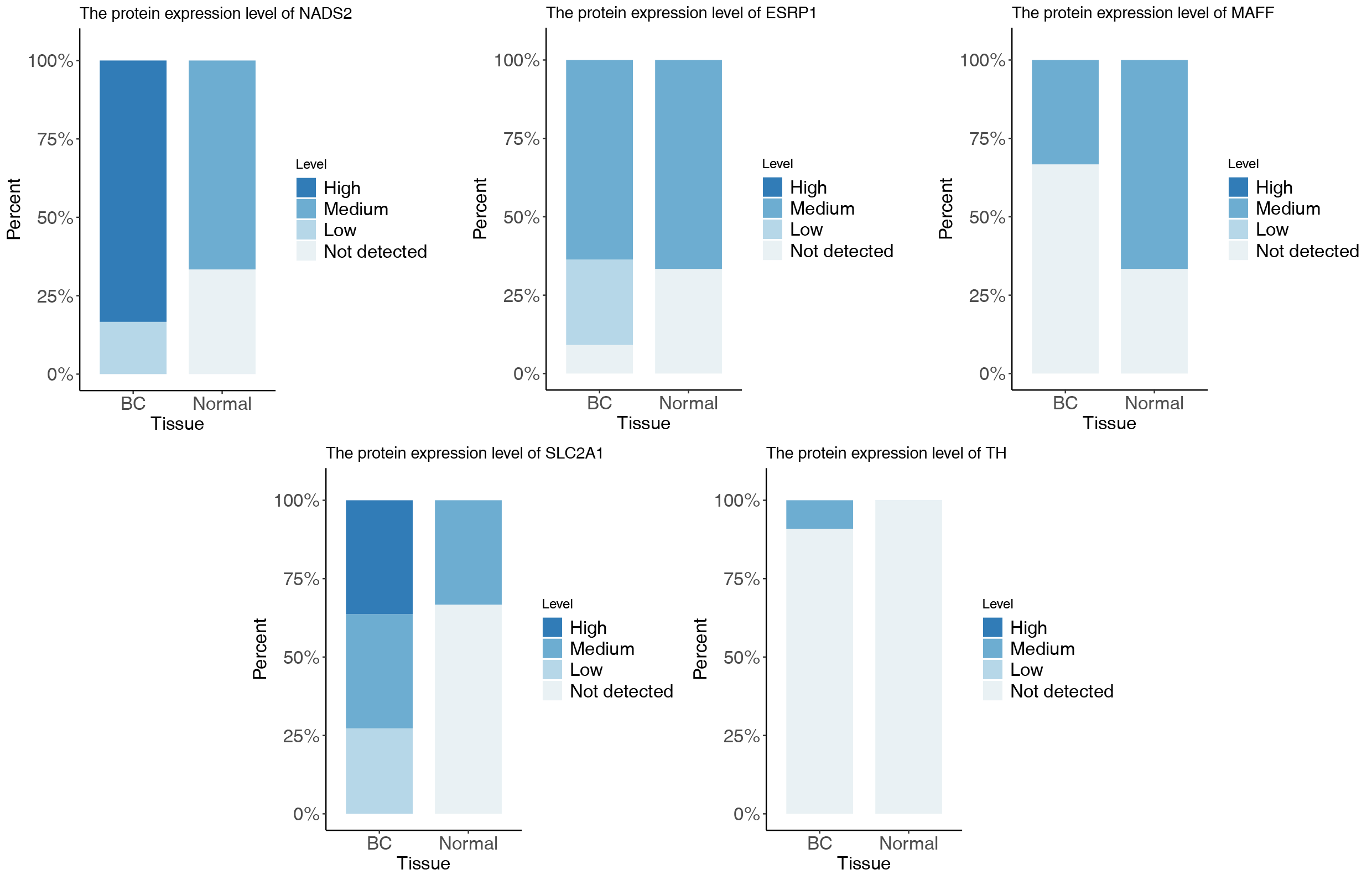

Supplement: Supplementary Figure 1 — The immunohistochemistry staining characteristics of the five HLRPGsfrom the HPA database. [file Image_1.tif]

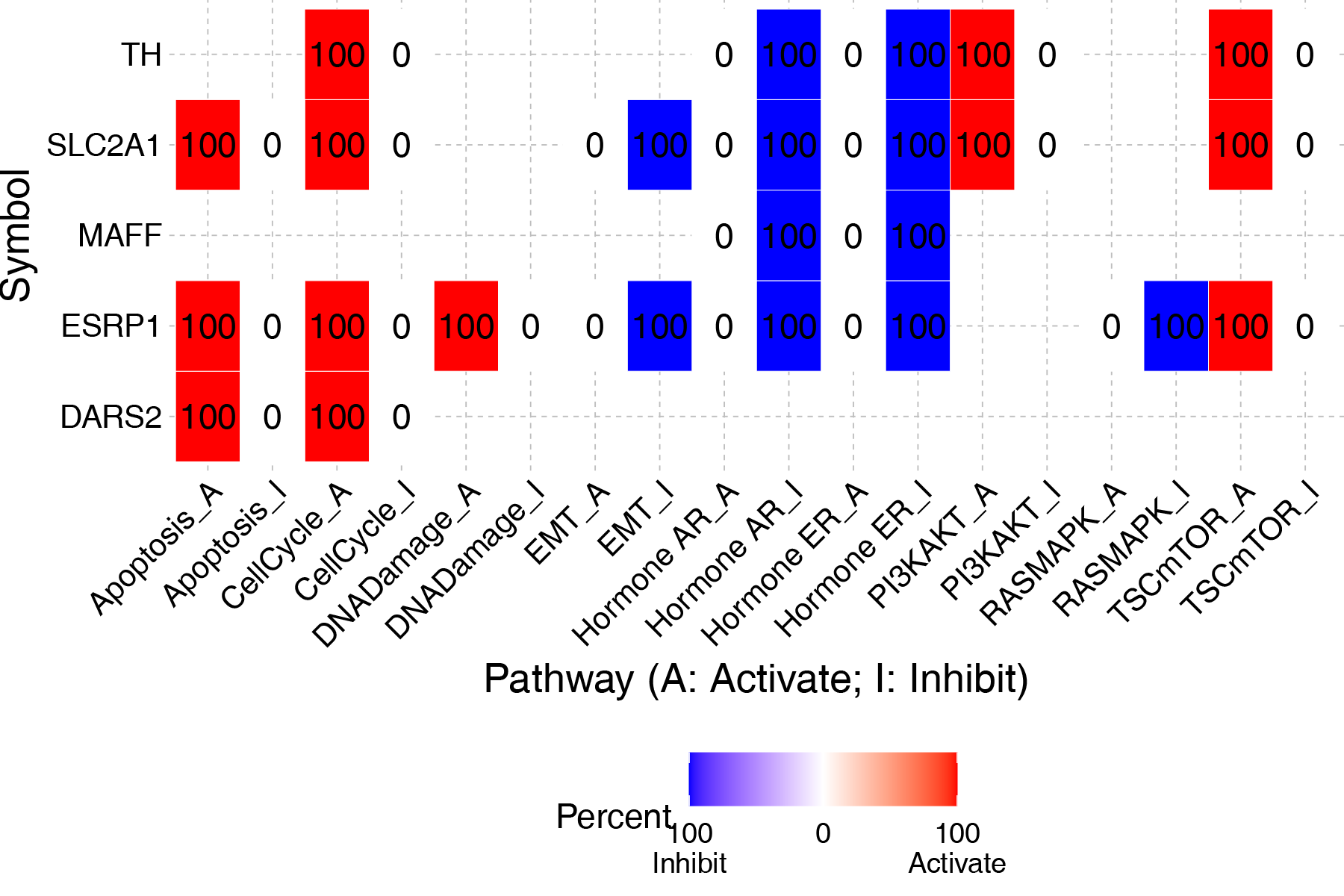

Supplement: Supplementary Figure 2 — The heatmap showed the correlation between five HLMRGs expressionsand the critical cancer signaling pathways. [file Image_2.tif]
